# Optimal duration of mechanical ventilation and influencing factors following mandibular distraction osteogenesis in infants with Pierre Robin sequence

**DOI:** 10.1097/MD.0000000000018339

**Published:** 2019-12-20

**Authors:** Na Zhang, Zhe Mao, Yingqiu Cui, Yonghong Tan, Huanhuan Zhang, Xiaoxin Ye, Yingyi Xu

**Affiliations:** a Department of Anaesthesia and Prioperative Medicine, Guangzhou Women and Children Medical Center; b Department of Stomatology, Guangzhou Women and Children Medical Center, Guangzhou, Guangdong, PR China; c University of New South Wales, Sydney, NSW, Australia.

**Keywords:** infant, mandibular distraction osteogenesis, mechanical ventilation, Pierre Robin sequence

## Abstract

Mandibular distraction osteogenesis (MDO) is an effective treatment for tongue-based airway obstruction in infants with severe Pierre Robin sequence (PRS). Most infants receiving MDO require postoperative mechanical ventilation (MV) to assist breathing. Optimal MV time for each individual patient and factors influencing the time must be identified to guide clinical decision-making.

A retrospective analysis was performed on 75 infants with PRS receiving MDO from November 2016 to August 2018. Twenty-six were females and 47 were males. Data extracted from the hospital information system included sex, age, weight, history of preterm labor, preoperative pulmonary infection, laryngomalacia/tracheomalacia, laryngoscope exposure classification, anesthesia duration, operation duration, postoperative treatment site, situation of distraction, postoperative complications and MV duration. Statistical analyses were conducted to investigate the potential associations of these factors with MV time.

Seventy-three PRS syndrome patients received anesthesia for MDO device procedures were considered eligible for study. Patient sex, history of preterm labor, preoperative pulmonary infection, laryngomalacia/tracheomalacia, laryngoscopy exposure difficulty, postoperative treatment site (neonatal or pediatric intensive care unit), ventilator-associated pneumonia, age, weight, anesthesia duration, and operation duration had no significant influence on postsurgical MV time (*P* > .05). Amount of distraction at the time of extubation had statistically significant influence on postoperative MV time (*P* < .05). In addition, scatter plots revealed linear relationships between postoperative MV time and amount of distraction at extubation.

According to this analysis, amount of distraction was associated with MV time following MDO for severe PRS and roughly 6 days post-surgery is a generally safe extubation time.

## 1 Introduction

Pierre Robin sequence (PRS), a congenital disease first described by French doctor Pierre Robin in 1923,^[1]^ is characterized by micrognathia, glossoptosis, and commonly a cleft palate. Many infants with PRS experience tongue-base airway obstruction and feeding intolerance due to micrognathia and glossoptosis, leading to hypoxemia, gastroesophageal reflux, malnutrition, and in severe cases to gradual weight loss, emaciation, and even death (mortality ranges from 1.7% to 65%).^[2–4]^

Up to 23% of infants born with PRS require surgical intervention due to severe airway obstruction.^[5]^ In recent years, mandibular distraction osteogenesis (MDO) has gained broad acceptance as an effective method to treat airway obstruction caused by PRS^[6]^; however, the majority of infants with MDO require mechanical ventilation (MV) to assist breathing in the postoperative period.^[7]^ The appropriate MV time is critical. If extubation is conducted too early, acute postoperative airway obstruction may occur, resulting in hypoxia, negative pressure pulmonary edema, increased incidence of secondary intubation. On the contrary, excessive MV time can increase the risks of other pulmonary complications,^[8]^ such as tracheal stenosis.^[9]^ Although a few studies^[10–13]^ have examined the duration of intubation after MDO, the optimal time for individual patients remains unclear due to differences in research populations, anesthesia protocols, and surgical methods. In addition, there have been few evidence-based analyses on the influences of various clinical and demographic factors on postoperative MV duration. Therefore, in this retrospective study, the optimal duration of intubation after MDO and multiple factors potentially associated with MV duration were analyzed to provide a foundation for decisions on safe withdrawal of MV for individual patients.

## 2 Methods

### 2.1 Study design

This non-randomized, retrospective single-center study included patients with PRS who underwent MDO at Guangzhou Women and Children's Medical Center from November 2016 to August 2018. Patients were excluded for missing case data. The study was approved by the Research Ethics Committee of the institution before data collection and written informed consent was obtained from all parents or guardians. The clinical trials have described in our manuscript been registered in China Clinical Trial Registry and the registration number was ChiCTR 1800018977. The informed consent of study was not given, because the design of the study was retrospective, and approval document of exemption from informed consent was uploaded to Trial Registry. Surgical options are mainly reserved for infants with severe airway obstruction who do not respond to non-invasive interventions, including prone positioning, application of nasopharyngeal airway, aimed at mitigating the need for tracheostomy. Severe airway obstruction is judged by oximetry criteria: clusters of desaturation with at least 3 dips <80 percentage,^[14]^ which was made in proximity of the surgical intervention. In addition, CT scan and fibrobronchoscopy were also performed to judge severe airway obstruction. Two reviewers independently identified subjects for inclusion and conducted data extraction.

### 2.2 Data collection

Demographic and clinical data collected included date of birth, sex, age, weight, gestational age, preexisting respiratory, and cardiac disorders, laryngomalacia/tracheomalacia, laryngoscope exposure classification, anesthesia duration, operation duration, situation of distraction, postoperative outcome, and postoperative complications (including ventilator-associated pneumonia, tracheal stenosis, required secondary tracheal intubation, and tracheostomy). Tracheomalacia: Characterized by an increased compliance of the intra or extra thoracic trachea resulting in dynamic collapse during the respiratory cycle.^[15]^ laryngomalacia: The diagnosis is confirmed by flexible laryngoscopy. Findings may include prolapse of the arytenoid cartilages or the supra-arytenoid mucosa, shortened aryepiglottic folds, and an “omega”-shaped or a retroflexed epiglottis.^[16]^ Preterm delivery was defined as gestational age less than 37 weeks. Difficult laryngoscopic exposure was defined according to direct laryngoscopic observation of glottis exposure on the anesthesia record sheet (Grade I/II was normal laryngoscope exposure, while grade III/IV was difficult laryngoscopy exposure). Ventilator-associated pneumonia diagnosis was defined by pneumonia occurrence 48 to 72 hours after tracheal intubation, new or advanced radiological infiltration, and one of the following 3 clinical features: temperature >38°C, white blood cells increase or decrease, and respiratory purulent secretion.

### 2.3 Surgical and traction procedures

**Figure 1:**
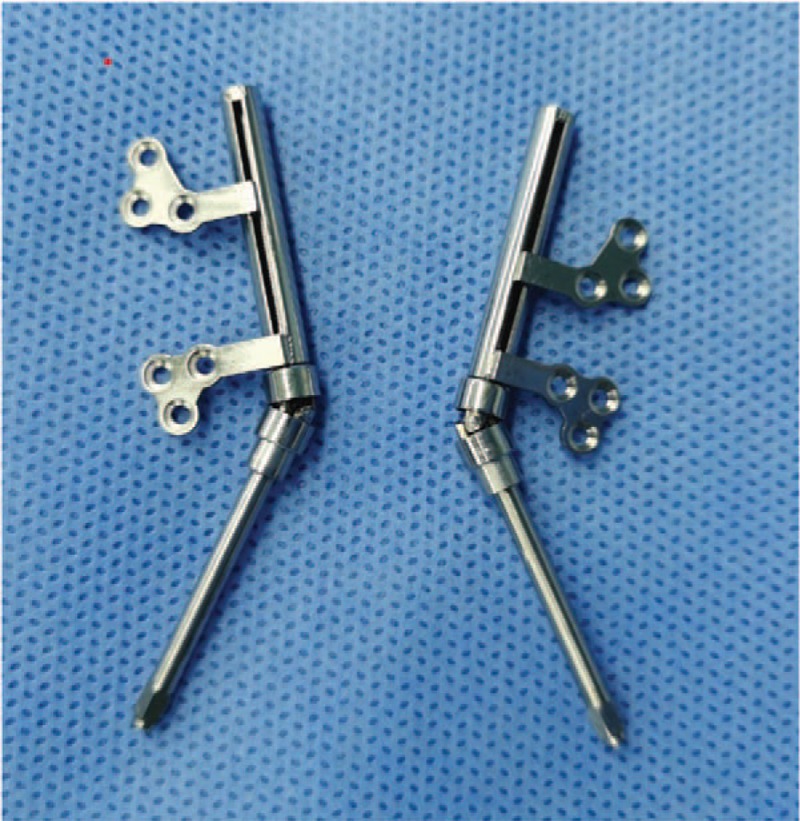
The distraction device.

The operations were performed by the same group of surgeons. There were 1 surgeon and 2 assistants performed the procedures (in the team) and they had performed mandibular distraction operation for more than 100 patients since 2016. Bilateral mandibular osteotomies were performed, and the distraction device secured with screws on either side. The selected traction device (10–20 mm; Zhongbang, Xian, China, Fig. 1) was placed at the mandibular angle. The direction of traction was along the condyle line to the mental point. Distraction activated 2.1 mm daily for 3 days (1.05 mm morning and 1.05 mm evening) and then 1.4 mm daily (0.7 mm morning and 0.7 mm evening) until the patient exhibited a crossbite of the anterior teeth. While the patient was mechanically ventilated at Intensive Care Unit, primary sedative agents were included fentanyl (2 mcg/kg/hour) and midazolam (0.1 mg/kg/hour), and Ramsay Sedation Score was maintained was at 3∼4. Midazolam and fentanyl infusions were discontinued at least 3 hours before planned extubation. Extubation timing is decided jointly by the intensive care and plastic surgery).

### 2.4 Statistical analysis

The study was a descriptive retrospective audit, and as such sample size estimation was not performed. All statistical analyses were performed using SPSS21.0. Kolmogorov-Smirnov test was used to assess normality of distribution of continuous data. Continuous data are expressed as mean ± standard deviation, and categorical data as frequency (percentage). Independent samples *t* test was used to compare MV duration between 2 groups defined by various parameter cut-off values, and Pearson correlation tests were used to identify factors influencing MV duration. All statistical analyses were two-tailed, and a *P* value less than .05 was considered significant.

## 3 Results

Seventy-five PRS syndrome patients received anesthesia for MDO device procedures, and 73 were considered eligible for study. On average, the infants were ventilated for 138.5 hours postoperatively (range: 41–357 hours, SD: 60.9 hours). The amount of distraction required for these patients ranged between 12 and 20 mm (mean was 15 mm) and days of distraction did patients get ranged between 5 and 11 (mean was 7).

Unexpected detachment of tracheal intubation occurred in 1 patient 2 days post-surgery, and tracheal re-intubation could not be performed due to respiratory conditions. There were no mortalities in this series. Four patients required a second intubation, 1 received tracheostomy for life-threatening upper airway obstruction, and 2 patients developed tracheal stenosis. Three patients needed continuous positive airway pressure after subject had been weaned to room air. Demographics and other clinic data are presented in Table 1.

**Table 1:**
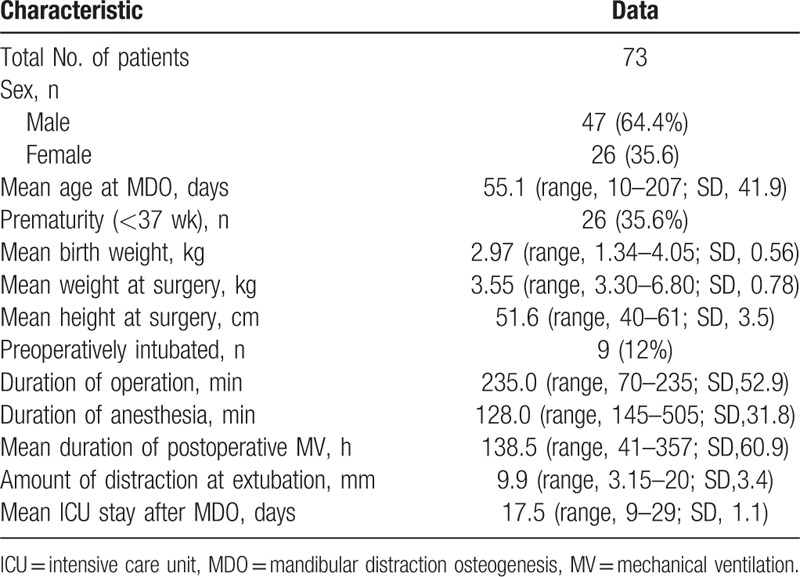
Demographics characteristics of patients.

**Figure 2:**
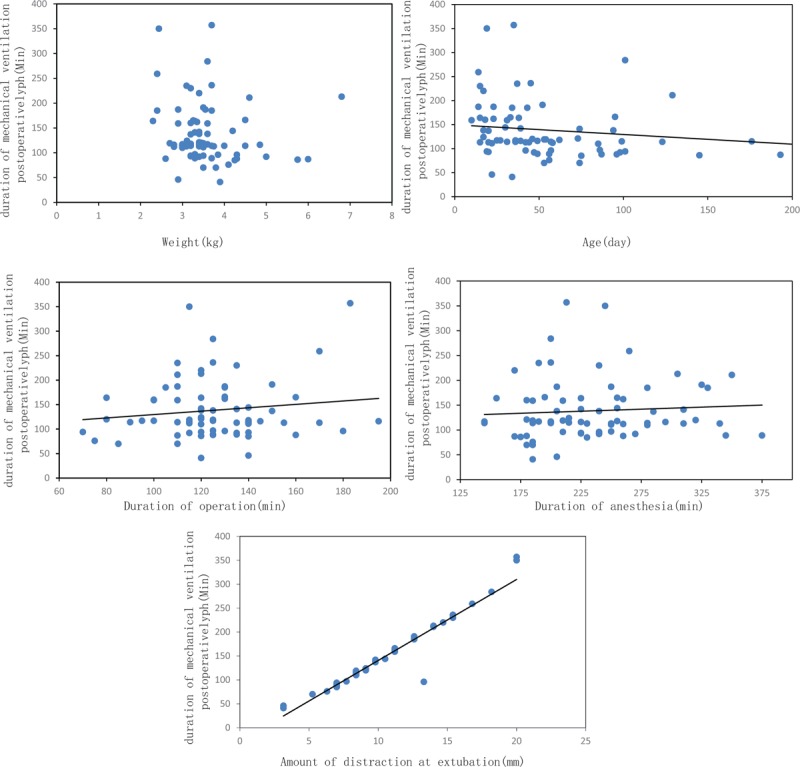
Scatter plots of selected continuous variables and postoperative MV time.

The influences of various binary variables on postsurgical MV time are shown in Table 2. Patient sex, history of preterm labor, preoperative pulmonary infection, laryngomalacia/tracheomalacia, laryngoscopy exposure difficulty, postoperative treatment site (neonatal or pediatric intensive care unit), and ventilator-associated pneumonia had no significant influence on postsurgical MV time (*P* > .05). The influences of continuous variables on postsurgical MV time are shown in Table 3. Age, weight, anesthesia duration, and operation duration had no statistically significant influence on postoperative MV time (*P* > .05). Amount of distraction at the time of extubation had statistically significant influence on postoperative MV time (*P* < .05). In addition, scatter plots revealed linear relationships between postoperative MV time and amount of distraction at extubation (Fig. 2).

**Table 2:**
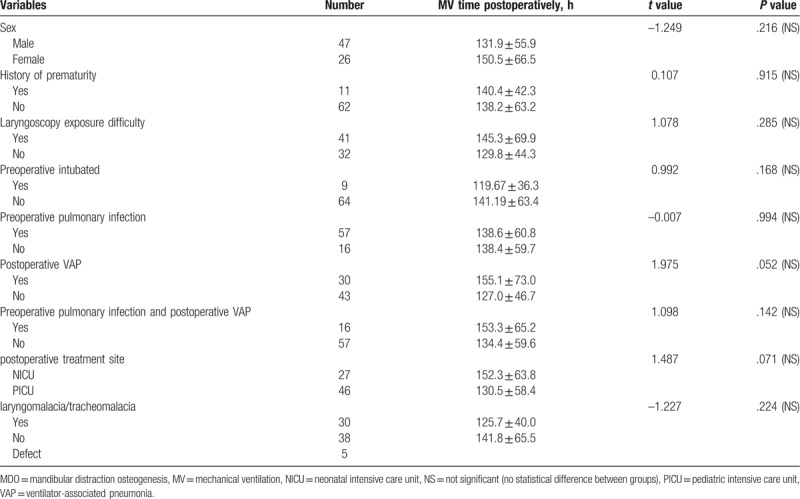
Influences of selected binary variables on postsurgical MV time.

**Table 3:**
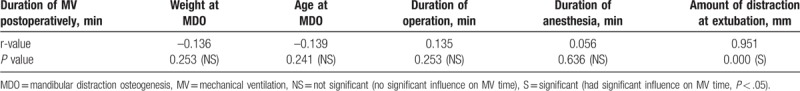
Influences of selected continuous variables on postsurgical MV time.

## 4 Discussion

In our study, 72 of 73 cases were extubated successfully and only 1 patient required tracheotomy. This proportion was less than others,^[17,18]^ in those studies, 3% to 8.5% of patients required postoperative tracheotomy because of unextubated. A report showed that central nervous system abnormalities, gastroesophageal reflux, airway abnormalities, cardiovascular complications, distraction longer than 30 days, and preoperative endotracheal intubation were potential risk factors with extubation difficulty.^[19]^ Our vast majority of infants had only minor atrial/ventricular septal defect, patent ductus arteriosus, and patent foramen ovale. In addition, fiberoptic bronchoscopy excluded possible other airway problems before distraction, and abnormalities of the central nervous system were presumably screened by preoperative examination. Therefore, these problems had not affected extubation in our infants.

We found that laryngomalacia/tracheomalacia did not affect postoperative MV duration, possibly because this factor was defined binary variables (Yes or No). Whether the degree of laryngomalacia/tracheomalacia affects MV time requires further investigation. Besides, preoperatively pulmonary infections and postoperative VAPs were extremely common (78% and 41%, respectively). A previous study^[20]^ found that preoperative pulmonary infection can prolong MV time following various types of surgery. However, we found no such association, possibly because we used lung-protective ventilation strategies during the operation.^[21,22]^ Furthermore, use of antibiotics and hormones accelerated improvement of pneumonia, so pulmonary infection was not severe enough to cause extubation failure. Although MV duration was longer in cases with postoperative ventilator-associated pneumonia, the difference did not reach statistical significance due to the small number of cases. Both conditions could have had a strong impact on the clinical course of recruited patients, but the analysis showed that it was not associated with a more severe course. This may be because there were not so many patients with both conditions. Therefore, additional studies with larger case numbers are required to determine the impact of Preoperatively pulmonary infections and postoperative VAPs on MV time.

This analysis found that amount of distraction was the only factor associated with MV time after MDO. Yin et al^[23]^ found that preoperative clinical symptoms associated with PRS, such as severe dyspnea, low body weight, and preoperative laryngoscope exposure difficulty were associated with dyspnea or blood oxygen saturation desaturation after surgery, which in turn led to later tracheal extubation. This may be because mandibular traction in their patients was not initiated at the time of extubation, and dyspnea and difficult airway were not resolved. In contrast, distraction have started in our infants at the time of extubation, and we speculate that preoperative airway obstruction and factors predisposing to intubation/extubation difficulty were gradually alleviated by extension of the mandible. MDO relieves airway obstruction by lengthening the mandible. This stretches the tongue attachments to the mandible (genioglossus muscle), which positions the tongue more anteriorly, relieving the glossoptosis.^[24,25]^ In previous studies, cephalometric analysis before and after mandibular distraction for congenital micrognathia revealed normalization of the maxillary-mandibular relationship and a mean increase in the cross-sectional airway area of 67.5%,^[26]^ and 3- to 20-fold increase in the distance from the post-pharyngeal wall to the lingual root.^[27]^ Computed tomography revealed a 200% increase in the cross-sectional area of the retroglossal oropharynx in 13 children.^[28]^ In addition, the airway tends to become more spherical.^[29]^ All of these changes mitigate respiratory difficulties caused by airway obstruction. On the other hand, a reduction in the incidence of difficult airway management from 71% to 8.3% was observed in infants with micrognathia following MDO.^[13]^ Difficult airway and airway obstruction were not the reasons restricting extubation as distraction proceeded. This means tracheal extubation can be performed when the patient has achieved proper distraction. Indeed, we thought that amounts of distraction expected was influenced MV time by the severity of micrognathia, is precisely because the severity of micrognathia. One would need different amounts of distraction in distinct patients to resolve dyspnea and difficult airway, anticipating longer courses (thus more prolonged MV) in those affected by the most severe forms of micrognathia. Of course, it would be more interesting to predict MV time by quantifying the severity of micrognathia.

Another finding of the present study is that optimal MV time was 6 days. Previous studies given wide-ranging results about airway management for infants after MDO and them lasted from 1 to 46 days.^[10–12]^ In general, surgically induced airway edema, trismus, and the need for analgesia during initial activation of the MDO device would appear to preclude earlier extubation. However, how many days of airway management for infants was needed to get through this period is no unified conclusion yet. In accordance with this finding, Geoff et al^[13]^ revealed similar time and they stated that a minimum of 5 days’ MV was most prudent considering patient age, history of preoperative upper airway obstruction and known difficult intubation. But they only speculated on this conclusion without detailed description and analysis.

This study has several limitations. First, PRS is a relatively uncommon disease, so it is difficult to collect large numbers of patients in a single medical center. Multi-center, large-sample studies are needed to verify these results, particularly regarding the influence of various rare preoperative conditions and postoperative complications. Second, the retrospective study design relies on accurate medical records, such as decisions regarding difficult airway were made subjectively according to the anesthesiologist's experience. Third, polysomnography study was not performed in enrolled patients and this could be a limitation.

## 5 Conclusion

In summary, amount of distraction was associated with MV time following MDO for severe PRS. Therefore, these infants can be performed extubation when they get appropriate distraction. This appears to be about 6 days for the typical patient.

## Author contributions

**Conceptualization:** Xiaoxin Ye.

**Data curation:** Zhe Mao, Huanhuan Zhang.

**Formal analysis:** Yonghong Tan.

**Funding acquisition:** Yingqiu Cui.

**Project administration:** Na Zhang, Yingyi Xu.

**Software:** Yingqiu Cui.

**Writing – original draft:** Na Zhang.

**Writing – review & editing:** Yingyi Xu.
